# Solid-State 3D Electrochemiluminescence Platform: Depth-Tuned Ru Complexes Positioning for Label-Free High-Resolution Imaging

**DOI:** 10.1021/acsomega.6c04224

**Published:** 2026-06-24

**Authors:** Chiara Mariani, Alessandro Auditore, Gabriele Giagu, Marta Penconi, Valentina Spampinato, Nunzio Tuccitto, Massimo Marcaccio, Giovanni Valenti, Alberto Bossi, Antonino Licciardello, Francesco Paolucci

**Affiliations:** † Department of Chemistry “Giacomo Ciamician”, Alma Mater Studiorum − University of Bologna, 40129 Bologna, Italy; ‡ Department of Chemical Sciences, Siciliae Studium Generale − University of Catania, 95125 Catania, Italy; § Istituto di Scienze e Tecnologie Chimiche“Giulio Natta” del Consiglio Nazionale delle Ricerche, CNR-SCITEC 20138,Milano, and SmartMatLab Centre, 20133 Milano, Italy; ∥ Center for Chemical Catalysis − C3, Alma Mater Studiorum − University of Bologna, 40129 Bologna, Italy; ⊥ CNR-ICMATE, Corso Stati Uniti 4, 35127 Padova, Italy

## Abstract

This study introduces a solid-state three-dimensional electrochemiluminescence (ECL) platform based on a mesoporous TiO_2_ superstructure deposited onto a fluorine-doped tin oxide (FTO) electrode and covalently functionalized with phosphonated [Ru­(bpy)_2_(Pbpy)]^2+^ complexes via zirconium phosphate (ZP) priming. By precisely controlling the vertical separation (L_2_) between immobilized luminophores and the underlying electrode, we uncover a depth-dependent ECL mechanism in which tripropylamine (TPrA) oxidation is driven selectively through a catalytic pathway. Time-of-flight secondary ion mass spectrometry (ToF-SIMS) confirms precise control over the vertical distribution of the luminophore, while electrochemical measurements reveal redox activity of immobilized Ru complexes mediated through the oxide scaffold. The platform further enables label-free shadow ECL imaging, in which localized perturbation of the catalytic ECL process generates high-contrast emission patterns. This architecture establishes a strategy for designing label-free ECL microscopy platforms featuring selective emission pathways and enhanced spatial resolution.

## Graphical abstract

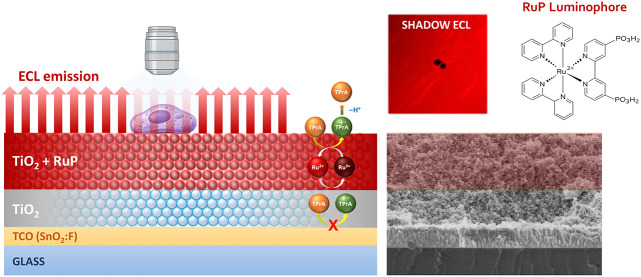

## Introduction

Electrochemiluminescence (ECL) is an advanced luminescence technique in which light is generated through electrochemically driven reactions, offering exceptionally low background noise, precise spatiotemporal control, high sensitivity, and a broad dynamic range.^1–4^ By decoupling electrochemical excitation from optical readout, ECL enables direct visualization of interfacial processes while providing mechanistic insight into signal-generation pathways.^5,6^

Among ECL systems, the tris­(2,2-bipyridyl) ruthenium (Ru­(bpy)_3_^2+^)/tri-n-propylamine (TPrA) pair remains a benchmark for both analytical and imaging applications^7,8^ due to its ability to regenerate the luminophore during the ECL process.^9^ In this system, ECL emission can proceed via multiple pathways, including oxidative-reduction, catalytic, and remote ECL routes,^4,10,11^ whose relative contributions depend critically on the concentration and spatial distribution of both the luminophore and the coreactant.^12,13^ Briefly, light emission originates from the formation of the excited state Ru­(bpy)_3_^2+^ through redox reactions between electrochemically generated Ru-based intermediates and TPrA-derived radicals. Depending on the operative pathway, both species are directly oxidized at the electrode, only the Ru complex undergoes electrochemical oxidation (catalytic route), or TPrA oxidation predominates at lower potentials. ECL microscopy (ECLM), particularly when coupled with three-dimensional (3D) engineered architectures, provides a powerful approach to interrogate these mechanisms and to selectively modulate emission pathways, with direct implications for imaging contrast and spatial resolution.^14,15^ While three-dimensional electrode architectures have been explored to enhance ECL performance,^16,17^ rigorous control of luminophore–electrode separation as a design parameter for regulating catalytic emission pathways has not been systematically investigated.

Here, we report the rational design and fabrication of a solid-state ECL platform based on a mesoporous TiO_2_ superstructure deposited onto a transparent FTO electrode by a doctor-blade printing approach.^18–21^ The TiO_2_ electrodes are further covalently functionalized with phosphonated ruthenium tris­(bipyridine) complexes, [Ru­(bpy)_2_(Pbpy)]^2+^ (RuP) (Figure 1a,b), using the zirconium phosphate priming strategy.^22–24^ By tuning the luminophore–electrode separation (L_2_) within the TiO_2_ scaffold, we selectively activate the catalytic ECL mechanism, where immobilized Ru complexes mediate TPrA oxidation at micrometer-scale distances from the underlying FTO substrate. This process is sustained by electronic mediation through the mesoporous oxide network rather than by direct electrode contact. The resulting ECL response varies predictably with luminophore confinement depth, while optimized potential windows preserve luminophore integrity over repeated cycles.

**Figure 1:**
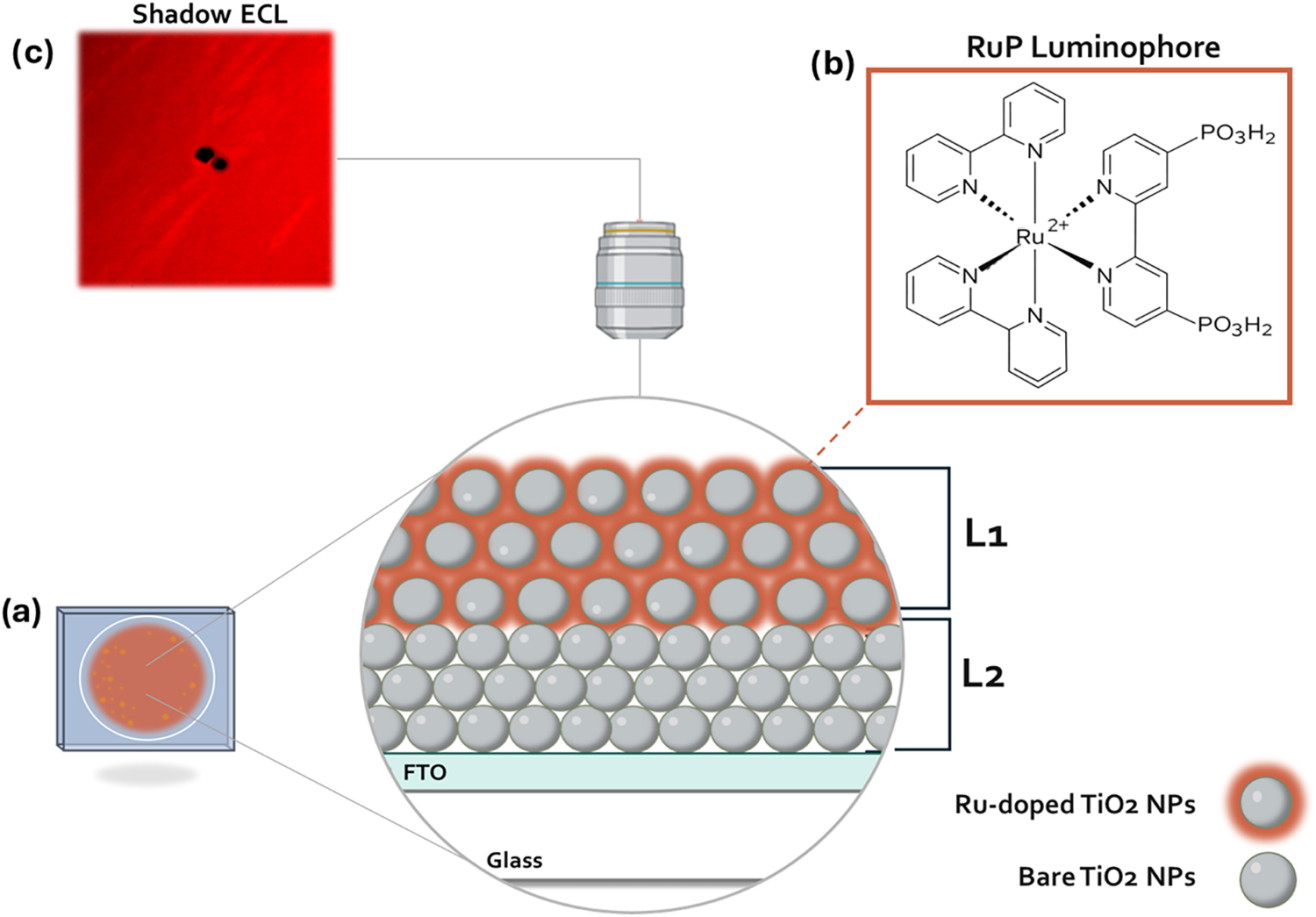
Schematic representation of shadow ECL imaging using a layered TiO_2_ architecture. (a) Cross-sectional view of the electrode, comprising a top layer of Ru-functionalized TiO_2_ nanoparticles (L_1_) and an underlying layer of bare TiO_2_ nanoparticles (L_2_) deposited on a FTO substrate supported by glass. (b) Structure of the immobilized phosphonated ruthenium complex (RuP). (c) Representative shadow ECL image acquired by ECL microscopy.

This architecture further enables label-free shadow ECL imaging (Figure 1c), in which localized perturbations of the catalytic ECL process yield high-contrast emission without requiring luminophore mobility. Collectively, these results establish a versatile solid-state ECL platform with enhanced control over emission pathways, spatial resolution, and imaging functionality.

## Materials and Methods

FTO coated glass slides, acetone (99.5%), isopropyl alcohol (99.8%) absolute ethanol (99.9%), phosphorus­(V) oxychloride (99%), zirconium­(IV) oxychloride hydrate (ZrOCl_2_·*x*H2O, 99.99%) were purchased from Sigma-Aldrich/Merck and used as received. TiO_2_ screen printing paste (Nanoxide T/SP, 100% anatase, average size 15–20 nm, surface area 100 m^2^g-^1^) was purchased from Solaronix SA. The water used in all experiments was prepurified using a Milli-Q (Millipore Corporation) device.

### Synthesis of Ru­(II) Bis­(2,2′-Bipyridine)- 4,4′-Bis­(Phosphonic Acid)-2,2′-Bipyridine [Ru­(bpy)_2_(Pbpy)]^2+^ (RuP)

The ruthenium complex was prepared according to the reported procedure.^25^ Cis-Bis­(2,2′-bipyridine)­dichlororuthenium­(II) dihydrate and [2,2′-Bipyridine]-4,4′-diyldiphosphonic acid were purchased from Fluorochem and used as received. Distilled water was further purified using a Milli-Q Ultrapure water purification system. To cis-Bis­(2,2′-bipyridine)­dichlororuthenium­(II) dihydrate (62.0 mg, 0.119 mmol, 1.00 equiv) dissolved in water (40 mL) [2,2′-Bipyridine]-4,4′-diyldiphosphonic acid (37.8 mg, 0.119 mmol, 1.0 equiv) was added and was heated under reflux for 18 h. After cooling to room temperature, the solvent was removed and an excess of acetone was added. The resulting solution was kept for 1 day at −12 °C. A precipitate formed, which was filtered and washed with cold acetone to give RuP as dark brown crystals (yield 86%). ^1^H NMR (400 MHz; DMSO-*d6*): 9.09 (d, 2H), 8.84 (d, 4H), 8.16 (q, 4H), 7.81 (m, 2H), 7.76 (d, 2H), 7.70 (d, 2H), 7.62 (dd, 2H) 7.52 (q, 4H). High resolution ToF-SIMS spectra, obtained from a tiny amount of sample deposited as a submonolayer onto a silicon substrate, show the presence of the molecular ion and of large characteristic fragments, confirming the proposed structure (figure S1).

### Electrode Preparation

FTO-coated glass slides were cleaned by sonication in a 1:1 (V/V) mixture of acetone and isopropyl alcohol, followed by rinsing in absolute ethanol and drying under a nitrogen stream. A 10 mm-diameter circular layer of TiO_2_ paste was deposited onto FTO using the doctor blade technique, guided by a 50 μm-thick hard mask. The samples were initially dried at 180 °C for 30 min, followed by annealing in air at 500 °C for 1 h, (heating rate 50 °C/min). In addition to combusting the organic matrix, the thermal treatment promotes the sintering of TiO_2_ nanoparticles leading to the formation of a mesoporous layer. To mitigate thermal shock—known to induce delamination at the TiO_2_/FTO interface—the samples were gradually cooled. The thickness of the resulting mesoporous TiO_2_ layers, determined via stylus profilometry (KLA Tencor P-7 Stylus Profiler), ranged between 5.5 and 7.5 μm. The morphology and quality of the FTO-TiO_2_ interface were investigated via field emission scanning electron microscopy (Figure S2). After TiO_2_ deposition/sintering the samples were subjected to the ZP functionalization.^22–24,26^ The samples were exposed to O_2_ RF plasma treatment (March instrument Plasmod, 75W, 0.4 Torr, 5 min) to remove potential organic residues and they were immersed in ultrapure water (Milli-Q – Millipore Corporation), to promote surface hydroxylation. After a gentle drying on a hot plate at 120 °C under nitrogen flow, the substrates were immersed in pure phosphorus­(V) oxychloride (POCl_3_) for 1 h and rinsed with ultrapure water. The phosphorylated TiO_2_ films were subsequently immersed in an aqueous solution of ZrOCl_2_·*x*H_2_O (≈5·10^–3^ M, Sigma-Aldrich) for 15 min, resulting in the “ZP” functionalization of the TiO2 layer throughout its entire thickness. The samples were then sequentially rinsed with ultrapure water and absolute ethanol. Subsequently, ZP-modified electrodes were further functionalized by immersing them – without prior drying – in a ≈ 5·10^–4^ M solution of RuP complex for controlled periods. This treatment yielded a stratified architecture in which only a defined fraction of the TiO_2_ film thickness was decorated with the immobilized luminophore. As illustrated in Figure 1, this design produced a tunable gap of bare TiO_2_ (L_2_) separating the luminophore functionalized region (L_1_) from the underlying FTO substrate. By varying the immersion time, the thickness of RuP-modified TiO_2_ layer (L_1_), and, consequently, the extent of the intervening TiO_2_ gap (L_2_), could be flexibly modulated. Following the luminophore uptake, the samples were thoroughly rinsed in absolute ethanol and dried under nitrogen stream. The overall functionalization protocol is outlined in Figure S3.

### Electrode Characterization

TiO_2_ layer thickness was measured using a KLA-Tencor P-7 stylus profilometer. Scanning electron microscopy (SEM) images were acquired by a LEO Gemini 1530 field emission electron microscope. ToF-SIMS analyses were performed using an ION-TOF TOF-SIMS IV (IONTOF GmbH, Münster, Germany) equipped with a 25 kV Bi/Mn liquid metal ion source for analysis and a 10 kV Cs^+^ source for sputtering. Depth profiles were acquired in dual beam noninterlaced mode, alternating sputtering cycles (Cs^+^, 10 keV, 20 nA, raster area 80 × 80 μm^2^) with analysis cycles (Bi_3_^+^, 25 keV, 0.3 pA) raster-scanned over a 30 × 30 μm^2^ area, concentric to the sputter crater. No charge compensation was required. The depth scale was calibrated by measuring by stylus profilometry the depth of the crater obtained by stopping the sputtering at the TiO_2_/FTO interface, and assuming a constant sputter rate during the depth profiling.

### Electrochemical Measurements

Electrochemical characterization was performed using a Biologic SP300 potentiostat in a custom three-electrode cell with ZP–TiO_2_ electrodes as the working electrode, a platinum wire counter electrode, and a saturated Ag/AgCl reference electrode. Cyclic voltammetry (CV) was conducted from 0 to 2 V vs Ag/AgCl at 100 mV s^–1^ in 0.3 M phosphate buffer (pH 6.8) containing 180 mM TPrA as the coreactant. During CV measurements, the ECL signal was detected through the transparent working electrode using a photomultiplier tube (PMT) positioned beneath the cell. The setup was enclosed in a dark box to minimize ambient light interference. The PMT output was amplified and recorded simultaneously with current and potential. ECL imaging experiments were carried out in a Redoxme Raman cell using the same counter and reference electrodes. Imaging was performed with a Nikon epifluorescence microscope equipped with an EM-CCD camera (512 × 512 pixels). ECL emission was triggered by applying 1.3 V vs Ag/AgCl in 0.3 M phosphate buffer containing 180 mM TPrA. Images were acquired with 200 ms exposure time under dark conditions.

## Results and Discussion

RuP-functionalized TiO_2_ electrodes with controlled luminophore–electrode separation (L_2_) were characterized by ToF-SIMS depth profiling. Figure 2 presents the negative-ion depth profiles of two TiO_2_ samples subjected to identical ZP priming conditions but immersed in the RuP solution for different durations. Signals attributed to TiO_2_^–^ and SnO_2_F^–^ ions are related to the titanium dioxide matrix and the FTO substrate, respectively; ZrO^–^ ion is indicative of the ZP functionalization; Ru^–^ and CN^–^ signals are attributed to the presence of RuP complex. ZrO^–^ signal extends throughout the entire TiO_2_ layer and diminishes near the TiO_2_/FTO interface, marked by the sharp rise in the SnO_2_F^–^ signal.

**Figure 2:**
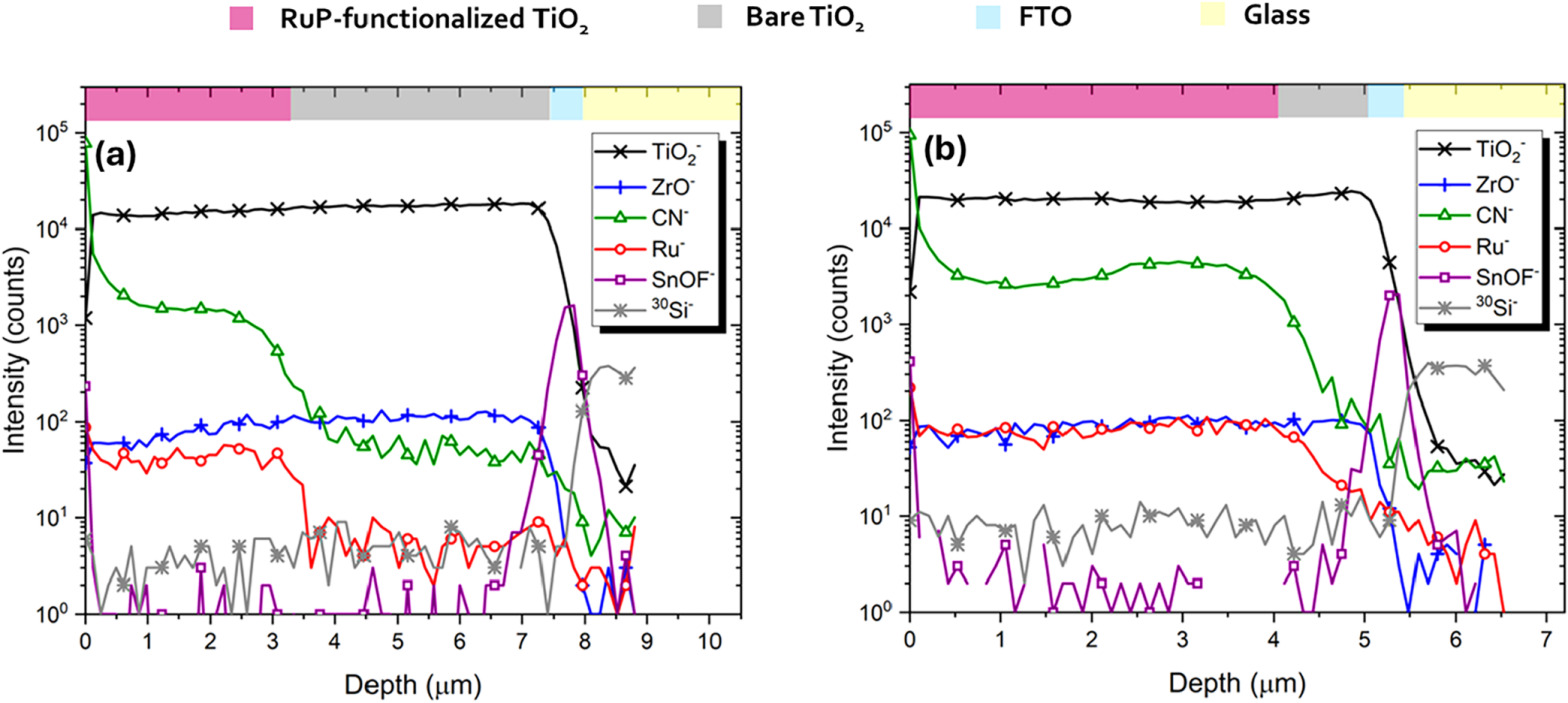
ToF-SIMS depth profiles acquired from mesoporous TiO_2_ layers exhibiting graded RuP functionalization, with total thicknesses of 3.2 μm (a) and 4.0 μm (b).

In contrast, the RuP luminophore functionalization is appreciably present only in the upper portion of the TiO_2_ film, with the functionalized thickness varying according to immersion time. In all cases, RuP-related signals (Ru^–^, CN^–^) diminish prior to reaching the TiO_2_/FTO interface.

Electrodes subjected to shorter immersion time show Ru^–^ and CN^–^ signals decay at approximately 2.1 μm from the surface (Figure 2a), whereas those treated for a longer duration exhibit RuP-related signals decay at a greater depth, around 4.4 μm. These findings confirm that the functionalization protocol enables precise control over the thickness of the RuP-functionalized layer (L_1_). Consequently, the thickness of the unmodified TiO_2_ region (L_2_), which separates the RuP-functionalized layer from the FTO interface, can be effectively tuned by varying the immersion time. Table 1 reports the L_1_ and L_2_ values, estimated by ToF-SIMS depth profiling, of four different samples with different values of L_2_, to investigate how the distance between the luminophore-containing layer and the FTO interface influences the ECL process.

**Table 1:** Thickness of the RuP-Functionalized TiO_2_ Layer (L_1_) and the Intervening Bare TiO_2_ Region (L_2_) for Representative Electrodes Prepared with Different Immersion Times, as Determined by ToF-SIMS Depth Profiling

SAMPLE	L_2_ (μm)	L_1_ (μm)
**A**	0.0	5.8
**B**	0.8	4.0
**C**	2.6	3.2
**D**	4.2	3.2

### Electrochemical and ECL Characterization

Cyclic voltammetry measurements reveal negligible redox activity for bare TiO_2_ electrodes in both PBS and TPrA (Figure 3a) indicating the absence of significant electrochemical processes under these conditions. In contrast, CV scans on RuP-functionalized electrodes show a sustained increase in current, particularly in the presence of TPrA (Figure 3a) and, importantly, an anodic process attributable to TPrA oxidation is observed even when the RuP complex is immobilized at approximately 1 μm from the electrode surface. Owing to its sluggish heterogeneous electron transfer kinetics, TPrA cannot be directly oxidized at the FTO surface under these conditions.^1^ The observed enhanced current response is therefore attributed to the redox mediation exerted by the confined Ru luminophore that sustains the catalytic oxidation of the coreactant (Figure 3b). Since simultaneous oxidation of both luminophore and coreactant is a prerequisite for the generation of the ECL signal (v. infra), the catalytic regime in these 3D porous electrodes necessarily involves anodic oxidation of the spatially confined Ru complexes, electronically mediated by the TiO_2_ nanoparticle network.^21,27–29^

**Figure 3:**
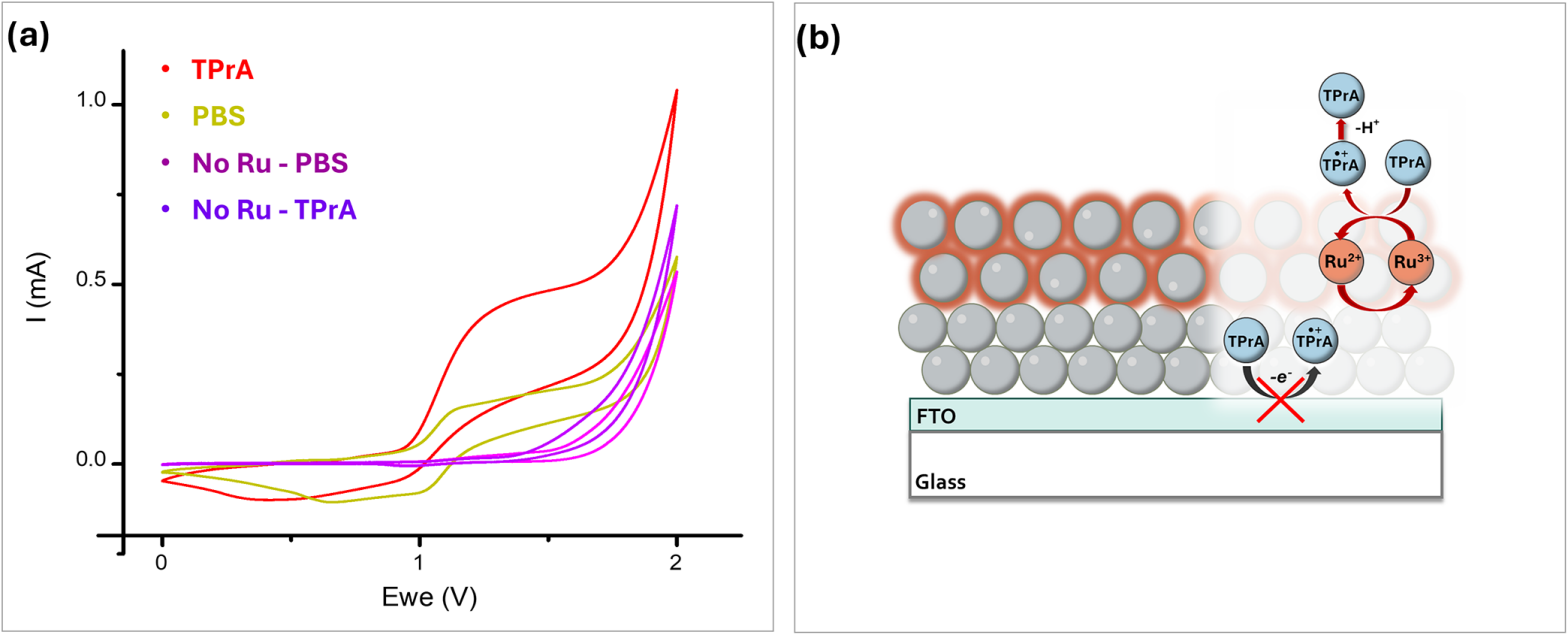
(a) Cyclic voltammograms of TiO_2_ electrodes with and without Ru functionalization in phosphate buffer and tripropylamine. (b) Schematic of the catalytic ECL pathway mediated by immobilized Ru^2+^/Ru^3+^ centers within the TiO_2_ matrix. Sample B, L_2_: 0.8 μm, L_1_: 4.0 μm.

To further probe this behavior, ECL profiles were acquired as a function of applied potential across electrodes with increasing L_2_, as detailed in Table 1. The data were normalized to emphasize differences in onset potential and emission shape (Figure 4). All ECL traces show a distinct emission located at early potentials, peaking at ∼1.2 V; however, its intensity significantly diminished as the parameter L_2_, i.e., the minimal distance of the luminophores from the electrode surface, increased. Electrode A (L_2_ = 0 μm; Figure 4, red curve), e.g., exhibits an early and broad ECL peak, characteristic of highly efficient catalytic activity. This is in line with the above hypothesis that TPrA oxidation is mediated by the oxidized Ru centers, electronically wired to the underlying FTO substrate through the TiO_2_ nanoparticles. As the separation distance increases, the gradual decrease in intensity suggests a growing impedance to electronic coupling between the confined Ru centers and the FTO substrate, resulting in less efficient oxidation of the luminophore.

**Figure 4:**
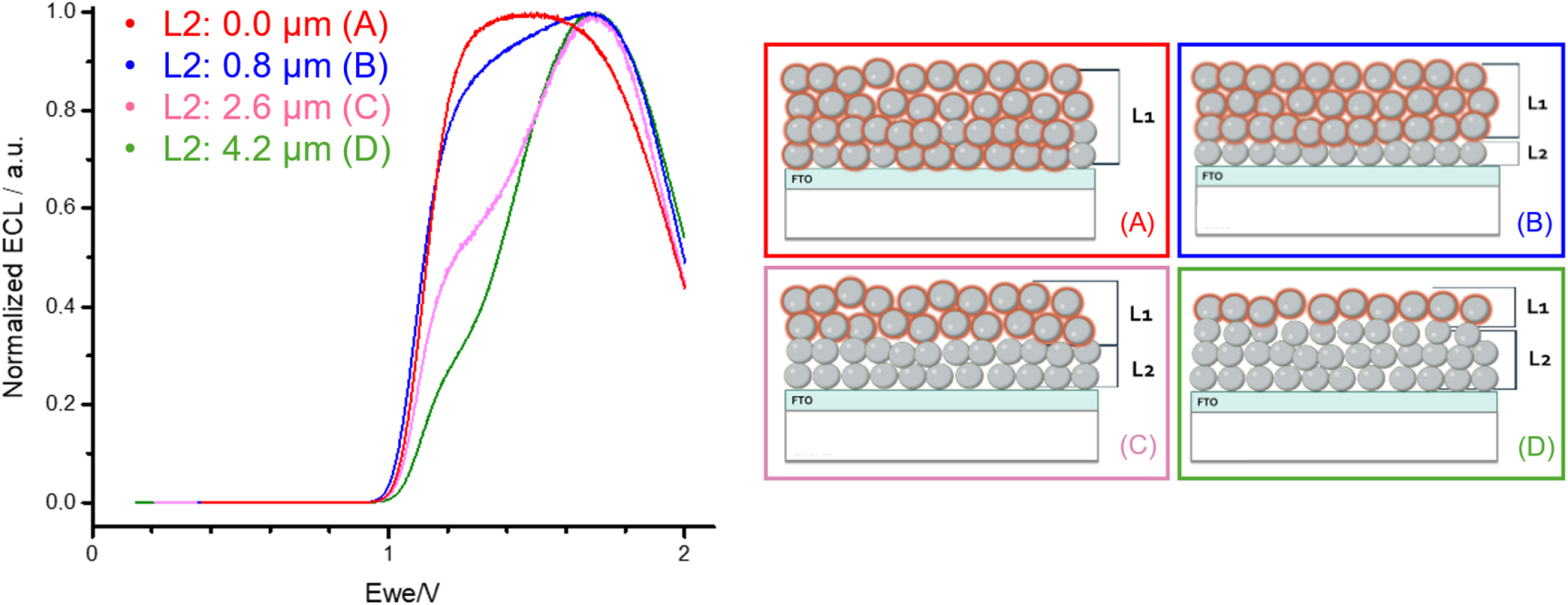
Normalized ECL intensity as a function of applied potential (E_we_) for electrodes with varying L_2_ layer thicknesses. Insets schematically illustrate the corresponding electrode architectures.

Although varying the immersion time also changes the thickness of the RuP-functionalized region (L_1_), the ECL behavior does not correlate solely with the amount of immobilized RuP. Instead, the relative contribution of the low- and high-potential ECL components evolves predominantly as a function of L_2_, indicating that luminophore–electrode separation plays a key role in determining the operative ECL pathway. At more positive potentials, all samples displayed a second emission, whose relative intensity increases with respect to the previous one as the separation between the luminophore and the electrode interface increases. While the original signal at ∼1.2 V is still clearly detectable in the intermediate samples, B (L_2_ = 0.8 μm; blue curve) and C (L_2_ = 2.6 μm; pink curve), it only appears as a minor shoulder on the main peak located at ∼1.7 V, for the deepest configuration L_2_ = 4.2 μm (sample D; green curve). To clarify the mechanism associated with the emission at higher potential, postoperando ToF-SIMS depth profiling was performed (Figure S4). After the application of high-potential conditions, a pronounced decrease in RuP-related secondary ion signals across the TiO_2_ layer is observed (Figure S4a), consistent with potential-induced desorption of the complex. When lower potentials are applied, instead, the RuP signal remains stable after ECL cycling, confirming that the luminophore remains anchored to the surface (Figure S4b). These findings suggest that, under operando conditions, partially desorbed and freely diffusing Ru species may activate a homogeneous catalytic pathway, in which TPrA oxidation is mediated by Ru centers that are first oxidized directly at the FTO surface. Such a hypothesis is also sustained by the CV data, whose evolution reflects a reduced catalytic efficiency of the TiO_2_-mediated oxidation with increasing luminophore–electrode separation (Figure S5).

These results strongly suggest that the high-potential emission component is consistent with homogeneous reactions involving TPrA,^30^ while limiting the potential within an optimized potential window preserves surface confinement and maintains a purely catalytic ECL regime. Accordingly, repeated CV–ECL cycles under controlled conditions, i.e., keeping the scan within 1.3 V, exhibit stable emission intensity (Figure S6), confirming the robustness of the immobilized luminophore.

Finally, to further support the proposed mechanistic hypothesis −that ECL generation at low potentials is predominantly governed by the conductive properties of the TiO_2_ scaffold – the normalized ECL curves in Figure 4 were deconvoluted into two asymmetric BiGaussian components (Figure S7). Notably, the intensity of the first component decreases inversely with increasing separation distance (L_2_), closely mirroring the trend observed for the CV current (Figure S5).

Overall, these findings highlight the RuP confinement depths (L_2_) as a key design parameter governing the efficiency of the catalytic ECL response. Greater distances from the electrode surface limit the local availability of RuP and its ability to oxidize TPrA efficiently. Conversely, when the Ru complex is positioned closer to the FTO interface, even within a few micrometers, it enables redox activation and ECL generation despite the absence of direct contact or diffusional mobility.

### ECL Imaging

Building on this mechanistic control, we further tested the nanostructured electrodes as novel platforms for SECL imaging. In contrast to homogeneous SECL systems,^31,32^ this platform employs an immobilized luminophore layer, while TPrA remains the only freely diffusing species. The immobilized Ru acts as a localized catalyst, driving TPrA oxidation to produce ECL.

To demonstrate this, a thin insulating ink layer was spray-deposited onto the TiO_2_ surface (sample B, L_2_ = 1 μm) to emulate biological objects that usually locally hinder coreactant diffusion. Upon addition of TPrA and application of an anodic potential (at 1.3 V vs Ag/AgCl, thus preserving the integrity of the immobilized RuP layer), ECL emission was generated uniformly across the electrode, with distinct dark regions appearing at locations where the catalytic ECL process was locally suppressed. Figure 5a shows the corresponding bright-field image, in which the deposited paint appears as a single spot. In contrast, the ECL image acquired with an EM-CCD camera (integration time: 6 s; Figure 5b) reveals two clearly distinguishable dark regions associated with the deposited insulating layer. Quantitative analysis of the extracted intensity profiles (Figure 5c) demonstrates that the ECL signal exhibits two partially resolved minima separated by approximately 15 μm, whereas the optical profile displays a single broad profile without resolved features. The observed contrast arises from localized suppression of the confined catalytic ECL process. While this behavior is consistent with reduced coreactant accessibility beneath the insulating layer, alternative contributions—such as modulation of the local electrochemical environment or altered oxidation efficiency of immobilized Ru centers—cannot be excluded.

**Figure 5:**
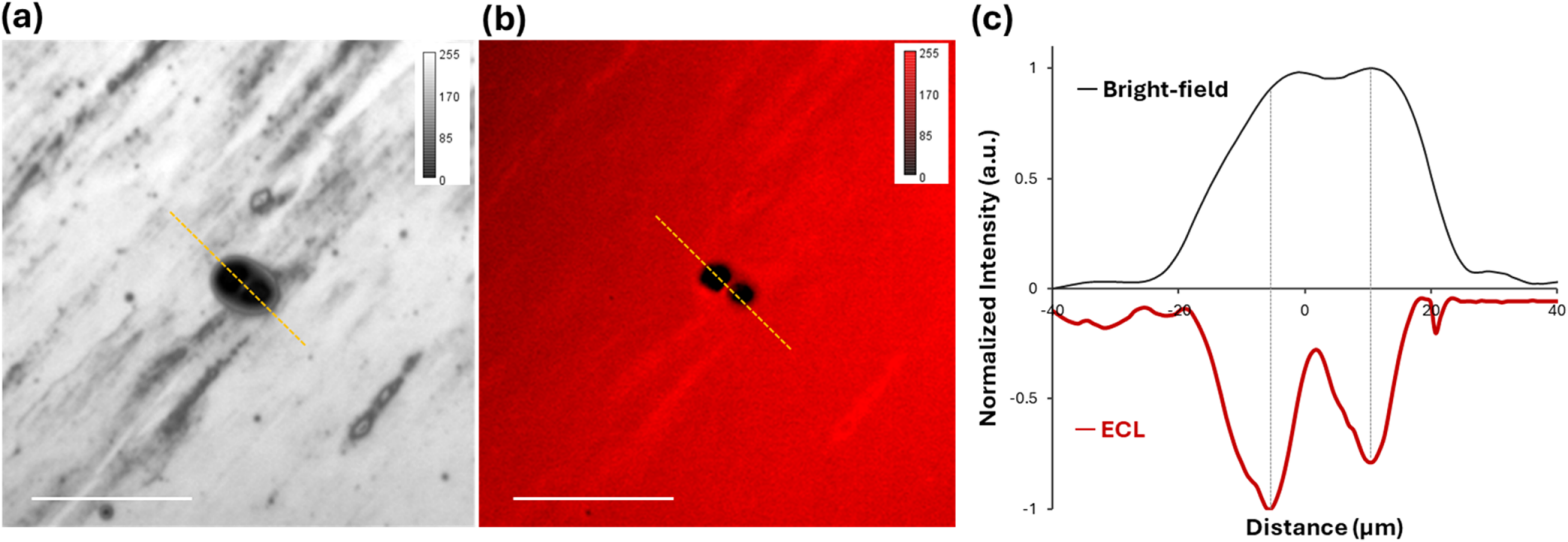
Bright-field optical image (a) and ECL imaging (b) of the sample surface, including the ECL intensity spatial profile (c). The ECL image was obtained with an EM-CCD camera by recording the ECL signal for 4 s during a two-step chronoamperometry measurement: 2 s at open circuit potential (OCP) and 4 s at 1.3 V vs Ag/AgCl. Magnification 20×; objective numerical aperture 0.4; gain 1; sensitivity, 100; Scale bar: 80 μm.

To assess system stability, we monitored ECL emission from this sample over time. The intensity remained nearly constant over 4 s of potential application (Supporting Movie 1), confirming the robustness of the immobilized Ru complex and the reproducibility of the SECL imaging platform.

## Conclusions

In this work, we developed a 3D ECL platform based on Ru-functionalized mesoporous TiO_2_ films, in which the spatial position of immobilized luminophores can be tuned with micrometric precision through a zirconium phosphate functionalization strategy. Unlike previously reported 3D ECL platforms, which primarily exploited nanostructuring to enhance signal intensity or increase active surface area,^16,17^ the present approach uses spatial confinement as a strategy to regulate the operative ECL pathway itself.^12^

ToF-SIMS depth profiling confirmed the formation of vertically graded RuP distributions within the TiO_2_ layer and enabled quantitative determination of both the functionalized region (L_1_) and the intervening bare TiO_2_ layer (L_2_). Systematic modulation of L_2_ revealed that the luminophore–electrode separation acts as a key structural parameter governing catalytic ECL efficiency. In particular, increasing the distance between the immobilized Ru centers and the FTO substrate progressively limits efficient electronic communication through the TiO_2_ scaffold, thereby modulating catalytic TPrA activation. These results demonstrate that catalytic ECL generation can be sustained by confined Ru centers electronically coupled to the underlying FTO substrate through the semiconducting oxide network.^21,28,29^

Beyond mechanistic investigation, this work establishes a new strategy for label-free shadow ECL imaging. In conventional shadow ECL microscopy, freely diffusing luminophores and coreactants generate emission near the electrode surface, while nonemissive objects locally hinder reagent accessibility to produce negative contrast.^31,33,34^ By contrast, the present architecture relies on immobilized Ru centers confined within the porous TiO_2_ matrix, while TPrA remains the only freely diffusing species. Consequently, localized perturbations of TPrA accessibility generate high-contrast emission patterns without requiring luminophore mobility while the imaged object remains electrochemically decoupled from the electrode surface.

Overall, this work identifies depth-controlled luminophore confinement as a new design parameter for engineering catalytic ECL processes in solid-state architectures. More broadly, the ability to spatially regulate ECL pathways through controlled luminophore positioning opens new opportunities for mechanistically tunable ECL platforms, spatially resolved electrochemical imaging, and advanced label-free microscopy strategies.

## Supplementary Material





## Notes

Time-dependent ECL emission monitoring system stability and robustness over potential application (Supporting Movie 1) (MP4)
ToF-SIMS spectrum of RuP luminophore (Figure S1); cross-sectional SEM images of mesoporous TiO_2_ layer after sintering (Figure S2); schematic sequence of operational steps for partially RuP-functionalized mesoporous film preparation (Figure S3); ToF-SIMS depth profiles of RuP on TiO_2_ before and after ECL analysis (Figure S4); cyclic voltammograms of Ru-functionalized TiO_2_ electrodes with varying TiO_2_ separation layer thicknesses (Figure S5); evolution of ECL signal and current profile as a function of applied potential (Figure S6); deconvolution of normalized ECL curves into asymmetric BiGaussian functions and Area2/Area1 ratio as a function of L_2_ (Figure S7) (PDF)
