# Supplementary material for: Solid-State 3D Electrochemiluminescence Platform: Depth-Tuned Ru Complexes Positioning for Label-Free High-Resolution Imaging
Source: ACS Omega. 2026 Jun 24;11(26):39371–8. doi: 10.1021/acsomega.6c04224 (PMC13347347; doi:10.1021/acsomega.6c04224)
Supplement: Supplementary file 2 [file ao6c04224_si_002.pdf]

Solid-State 3D Electrochemiluminescence Platform: Depth-Tuned Ru Complexes Positioning for Label-Free High-Resolution Imaging

Chiara Mariani,[‡,a] *Alessandro Auditore,**[‡,b,] *Gabriele Giagu,*[a] *Marta Penconi,*[c] *Valentina Spampinato,*[b] *Nunzio Tuccitto,*[b] *Massimo Marcaccio,*[a,d] *Giovanni Valenti,*[a,d] *Alberto Bossi,*[c] *Antonino Licciardello,*[b] *Francesco Paolucci.**[a,d,e]

[a] Department of Chemistry "Giacomo Ciamician", Alma Mater Studiorum − University of Bologna, 40129 Bologna, Italy
[b] Department of Chemical Sciences, Siciliae Studium Generale – University of Catania, 95125 Catania, Italy
[c] Istituto di Scienze e Tecnologie Chimiche "Giulio Natta" del Consiglio Nazionale delle Ricerche, CNR-SCITEC 20138, Milano, and SmartMatLab Centre, 20133, Milano, Italy
[d] Center for Chemical Catalysis − C3, Alma Mater Studiorum − University of Bologna, 40129 Bologna, Italy
[e] CNR-ICMATE, 35127 Padova, Italy

Supporting information

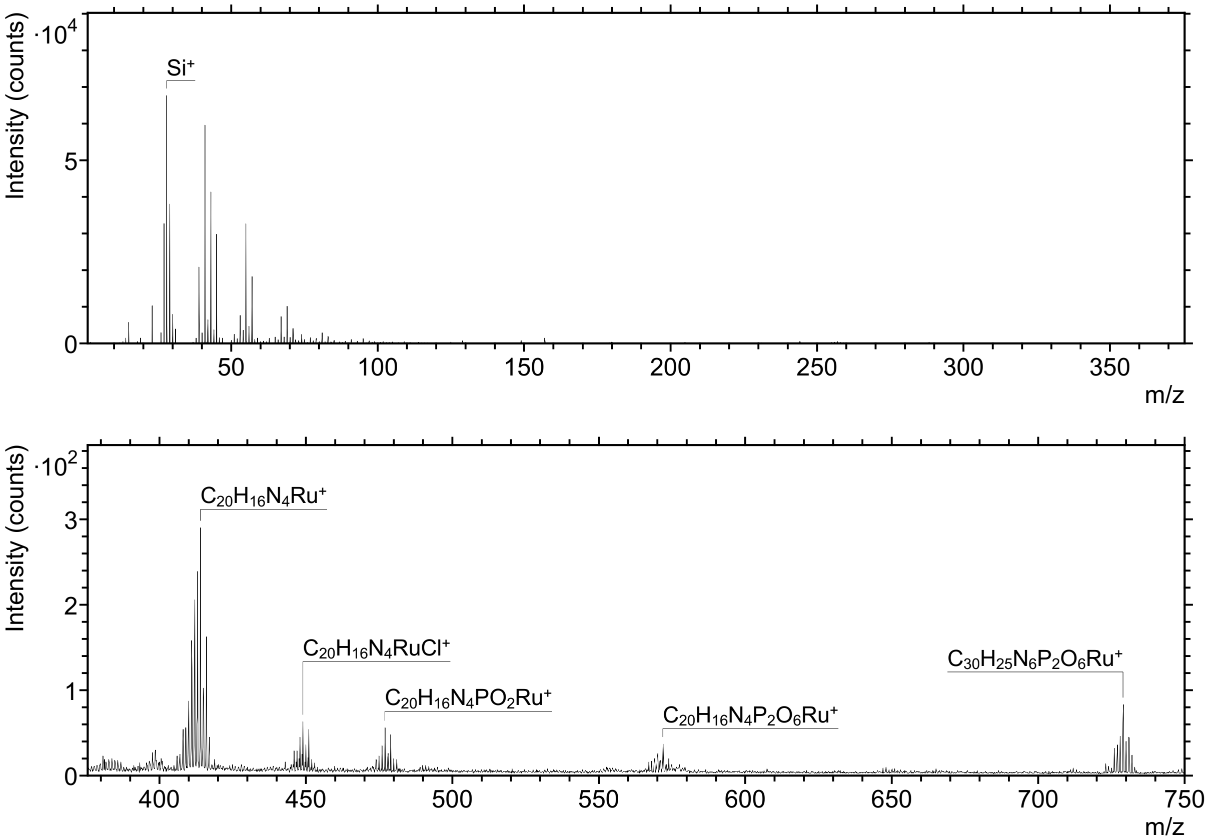

Figure S1: ToF-SIMS spectrum of RuP luminophore. Molecular ion ($C_{30}H_{25}N_6P_2O_6Ru^+$) and the most relevant fragment ions are highlighted and assigned by the corresponding peak labels.

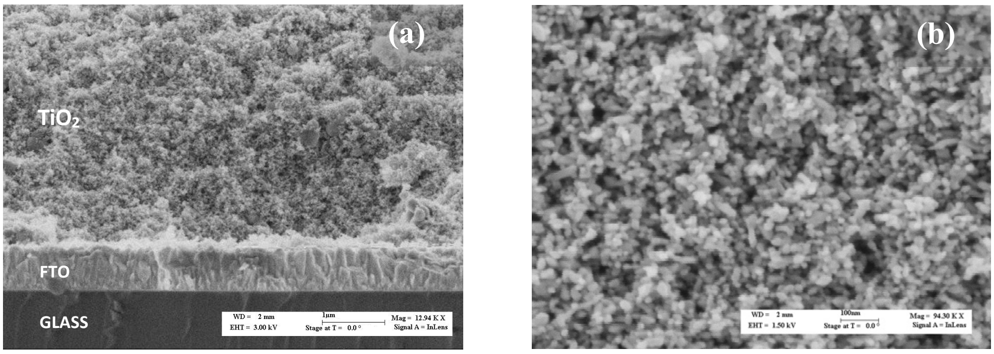

Figure S2: Cross-sectional SEM images of mesoporous TiO_2 layer after sintering process; FTO-TiO2 interfacial region (a) and mesoporous TiO2 structure (b).

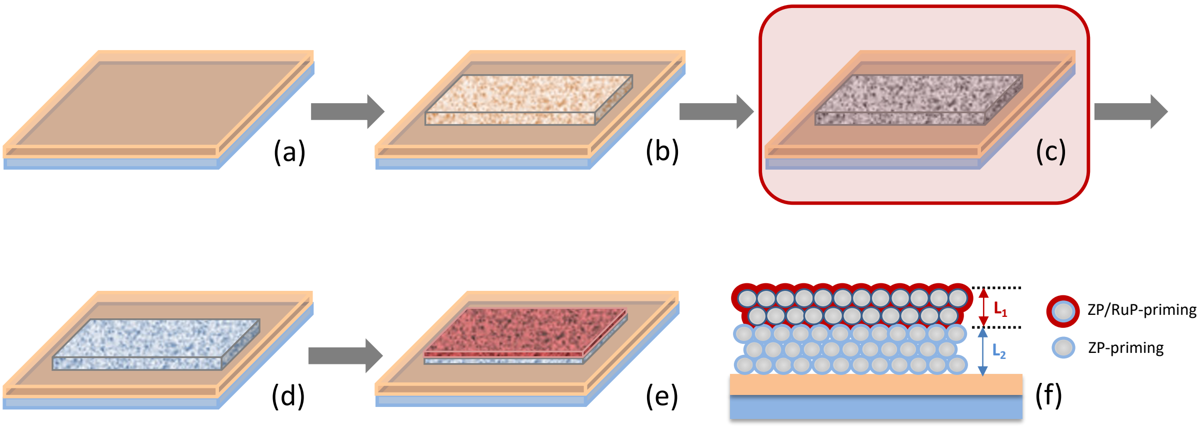

Figure S3: Schematic sequence of the operational steps for the preparation of a partially RuP-functionalized mesoporous film; **(a)** FTO-coated glass substrate; **(b)** screen-printing deposition of the paste; **(c)** thermal treatment at 500 °C; **(d)** ZP-priming process; **(e)** depth-selective RuP functionalization; **(f)** schematic representation of the final partially functionalized structure.

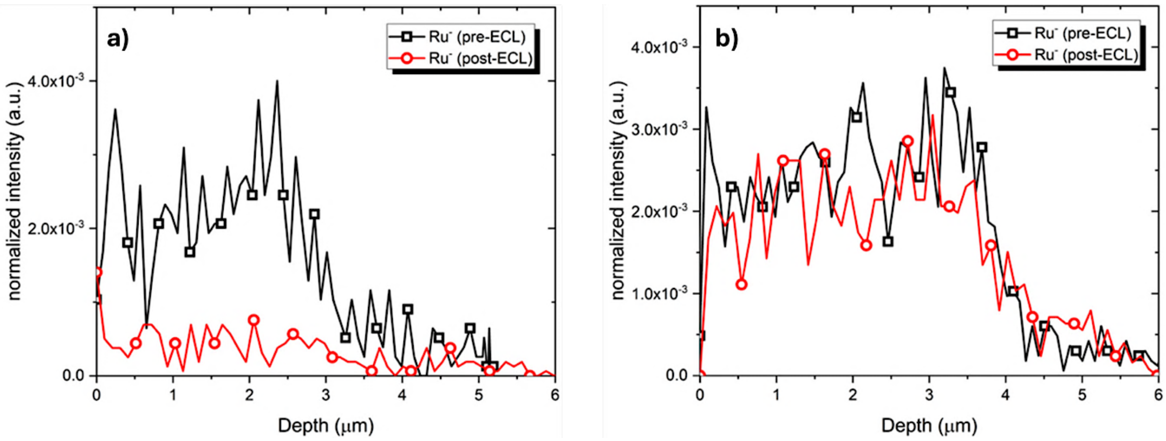

Figure S4: ToF-SIMS depth profiles of RuP on TiO_2 before and after ECL analysis. Ru- intensity is normalized to the TiO_2 signal. At high potential **(a)**, RuP-related signal decreases significantly post-ECL, indicating desorption. At low potential **(b)**, RuP-related signal remains stable, confirming retention of surface-bound RuP.

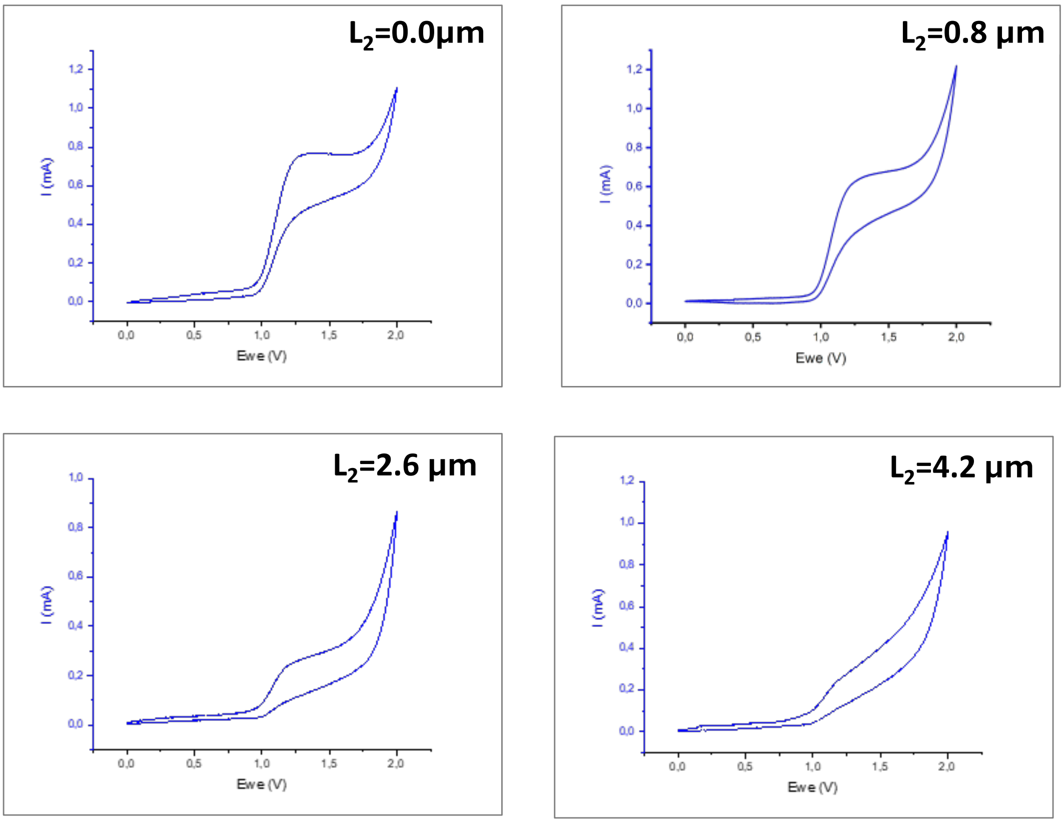

Figure S5: Cyclic voltammograms of Ru-functionalized TiO_2 electrodes with varying TiO_2 separation layer thicknesses (L_2 = 0.0, 1.0, 2.6, and 4.2 µm). The voltammograms were recorded in TPrA solution (180mM), PB 0.3M, pH 6.8, at v=100 mV s^{-1}.

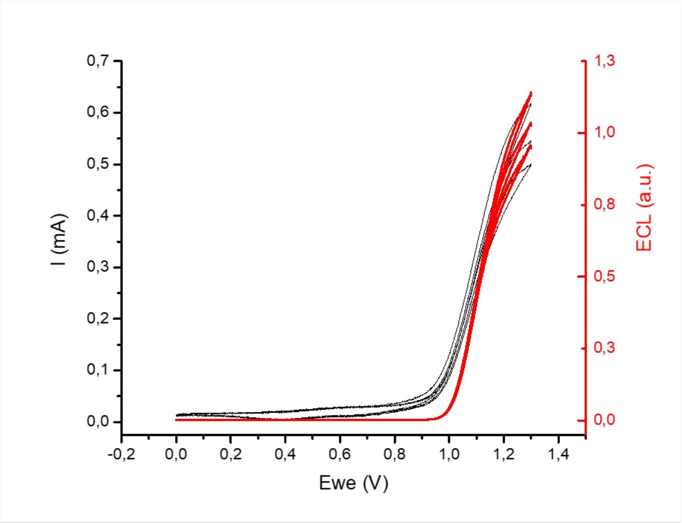

Figure S6: Evolution of the ECL signal (red) and current profile (black) as a function of applied potential (V vs. Ag/AgCl) across multiple scan cycles.

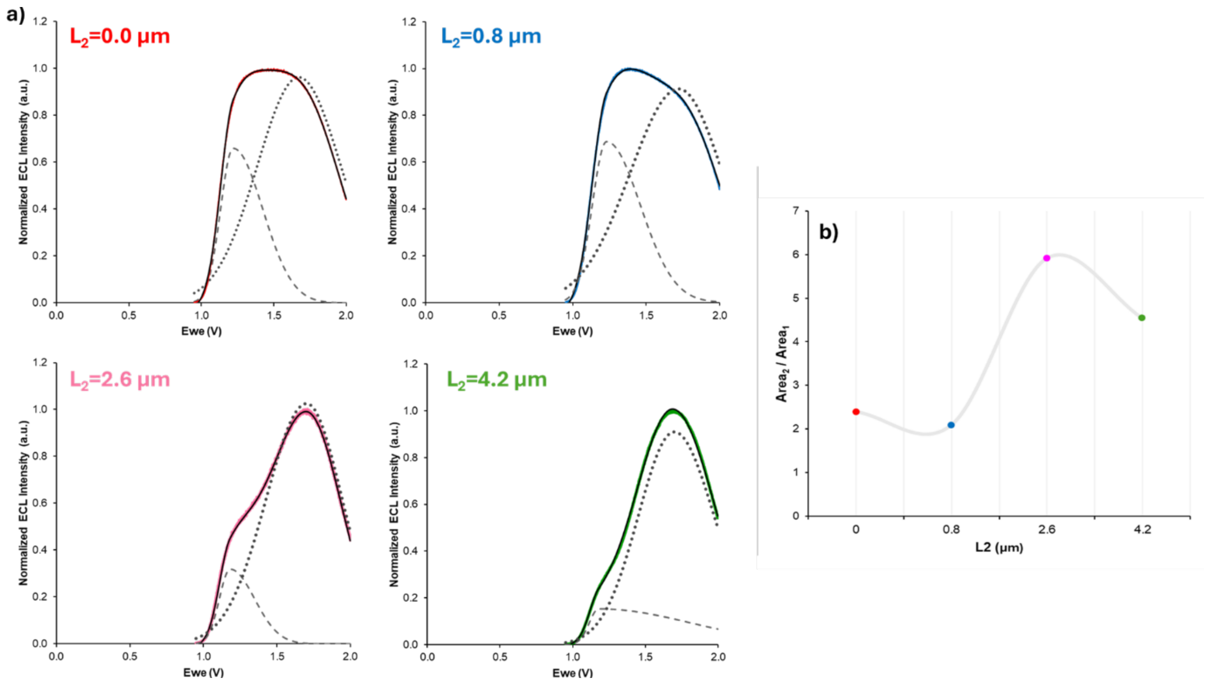

Figure S7: (a) Deconvolution of normalized ECL curves into two asymmetric BiGaussian functions for samples with increasing separation distance L_2 between the Ru-doped layer and the electrode interface: (A) $L_2 = 0$ µm, (B) $L_2 = 0.8$ µm, (C) $L_2 = 2.6$ µm, and (D) $L_2 = 4.2$ µm. Experimental data are shown as colored lines, the overall fit as a black line, and the two fitted contributions as gray dashed and dotted lines. **(b)** Ratio $Area_2/Area_1$ as a function of L_2, highlighting the increasing relative weight of the second contribution at larger separation distances.
